# Study on the effects of mental training on brain functional networks in operators during the glide phase of simulated carrier-based aircraft landing

**DOI:** 10.3389/fpsyg.2026.1798764

**Published:** 2026-04-07

**Authors:** Yipeng Chang, Jin Ma, Chaolin Teng, Wendong Hu, Lin Cong, Shan Cheng, Weitao Dang, Zijia Liu, Zongnan Zhang, Yiming Chen, Duoduo Hui

**Affiliations:** 1 Department of Aerospace Medicine, The Fourth Military Medical University, Xi'an, Shaanxi, China; 2 School of Basic Medical Sciences, The Fourth Military Medical University, Xi'an, Shaanxi, China

**Keywords:** brain functional network, EEG power spectral density, functional connectivity, mental training, sample entropy, simulated approach and landing

## Abstract

**Introduction:**

To enhance flight performance and safety margins during the glide phase of carrier-based aircraft landing, this study systematically investigated the effects of integrated mental training on trainees’ brain functional networks from four dimensions: flight performance, electroencephalogram (EEG) power spectral density, sample entropy, and functional connectivity.

**Methods:**

The study recruited 46 participants, divided into a training group and a control group. All participants wore EEG equipment and performed dynamic carrier-based landing tasks based on a simulated carrier aircraft approach and landing platform.

**Results:**

The results showed that, after mental training, the training group exhibited significantly better flight performance than before training, exhibiting reduced landing trajectory deviation and shorter flight durations, whereas no significant changes were observed in the control group. EEG analysis revealed that the training group exhibited significantly increased power spectral density in the alpha band of the frontal, parietal, and occipital regions, the theta band of the central region, and the beta band of the occipital region following training, indicating enhanced attention and working memory. Sample entropy analysis revealed increased theta-band complexity in the central and parietal regions of the training group, reflecting heightened brain activity in these areas. Functional connectivity analysis revealed enhanced connectivity between delta and alpha frequency bands in the training group, primarily involving collaboration between the frontal lobe and other regions.

**Discussion:**

In summary, the mental training paradigm employed in this study significantly enhanced the stability, precision, and efficiency of participants’ landing approach maneuvers. The underlying mechanism may be related to the optimization of brain functional networks. The research findings can provide reference for optimizing the carrier-based aircraft pilot landing training system.

## 1 Introduction

With high overload, extended flight duration and intense countermeasures becoming the norm in modern aerial combat, military pilots must not only endure immense physiological stress but also maintain high cognitive efficiency and emotional stability in an environment of information overload and rapidly changing battle situations. Consequently, mental training has become a subject of significant focus within the military domains of various nations. Such training enhances pilots’ perceptual abilities, attention span, spatial orientation skills, emotional control, and resilience under pressure (Manzey et al., 1995; Yanzeng et al., 2025). Many countries use mental training to enhance pilots’ mental resilience and ensure flight safety. A recent meta-analysis by Ming and Nargiza (2024) systematically evaluated the influence of mindfulness and mental resilience training on pilots’ cognitive performance and stress management, finding positive effects across various intervention approaches. Zhang et al. (2024) further demonstrated that a one-month Quick Coherence Technique breathing training programme improved pilots’ psychophysiological indicators associated with stress resilience and cognitive functions in both daily life and flight operation settings.

When an aircraft carrier navigates at sea level, it undergoes not only horizontal motion along the water surface but also rolling, pitching, and heaving motions in response to waves. Furthermore, atmospheric turbulence, upwash, and wake turbulence are present above the carrier (Bardera and Matias-Garcia, 2022; Zamiri and Chung, 2025; Duan et al., 2022). Therefore, compared to land-based operations, carrier-based aircraft face a more complex operational environment with significantly heightened risk factors. Furthermore, the aircraft carrier’s flight deck presents a three-dimensional, non-linear motion pattern spanning approximately 200 metres in length. This characteristic renders take-off and landing the most challenging and hazardous phases of carrier-based aircraft operations (Tkachenko et al., 2019; Liu et al., 2023; Liu and Wang, 2025). Zhu et al. (2025) demonstrated that pilots’ mental workload during the landing glide phase is significantly higher than in other phases of carrier-based flight operations, underscoring the critical importance of this phase for flight safety. The landing glide phase not only carries the heaviest operational load but also determines whether an escape and go-around manoeuvre is executed, making it critically important for flight safety.

Carrier-based aircraft pilots must possess superior physical and psychological fitness, adaptability and responsiveness, deception resistance, and flight handling skills (Hui Li et al., 2015). Relevant research indicates that systematic mental training can significantly enhance pilots’ perceptual acuity, attention allocation efficiency, spatial orientation precision, emotional regulation capabilities, and stress resilience, directly translating into improved operational performance and combat safety factors (Liu et al., 2023). However, the question of whether systematic mental training can enhance immediate performance on complex tasks through neuroplasticity mechanisms remains largely unexplored (Ehrhardt et al., 2024; Zhang et al., 2025). Therefore, investigating the transfer effects of mental training during this critical phase and its underlying neural mechanisms holds significant theoretical and practical implications for enhancing the operational proficiency of carrier-based aircraft pilots.

In recent years, advances in non-invasive brain function monitoring technology have provided a reliable means of revealing the immediate and sustained effects of mental training. Electroencephalography (EEG), as one of the commonly used brain imaging techniques, directly reflects the neural functional state of the brain. It offers advantages such as being non-invasive, possessing high temporal resolution, being convenient to acquire, simple to operate, and relatively inexpensive. Consequently, it is widely employed by researchers in the study of brain functional activity (Hramov et al., 2021; Värbu et al., 2022; Hassan et al., 2025; Hussein et al., 2023). EEG enables millisecond-level real-time observation of brain activity, providing a direct measurement of cognitive load responses within the central nervous system. Currently, numerous studies have employed EEG for monitoring and assessing cognitive load in drivers and pilots (Yoo et al., 2023; Zhou et al., 2025; Feltman et al., 2024). Moreover, extensive research has demonstrated that specific frequency bands within EEG signals—such as Alpha, Beta, Theta, and Gamma—are closely associated with psychological processes including attention, memory, and cognitive control. These processes, in turn, significantly influence an individual’s reaction speed across a variety of tasks (Klimesch, 1999; Gola et al., 2013; Cavanagh and Frank, 2014; Diaz-Piedra et al., 2020).

Ji et al. (2024) employed the beta wave power ratio and Shannon entropy as EEG features to quantitatively classify pilots’ mental workload during different phases of simulated flight operations, finding that both metrics exhibited significant alterations during left and right turns compared to the cruise phase. Binias et al. (2023) investigated the relationship between pilots’ attention and reaction times during short simulated flights and specific brain activity, finding a significant positive correlation between theta power in the frontal lobe and reaction time duration. Feltman et al. (2021) found that frontal alpha and beta waves correlate with perceived workload during auditory tasks, while frontal theta waves are associated with perceived workload during visual tasks. Zhang et al. (2022) employed EEG analysis during flight missions to examine pilots’ brainwave indicators sensitive to error awareness both on the ground and in flight, thereby identifying differences in the EEG characteristics associated with error awareness between these two environments. Additionally, research has employed neurobiological models based on functional connectivity in EEG to identify hazardous flying behaviours among carrier-based aircraft pilots (Li et al., 2023).

However, the aforementioned studies have predominantly focused on static or steady-state flight missions, and have not yet addressed the high-time-varying, high-risk glide phase of the carrier-based aircraft landing. Moreover, according to our best knowledge, there is currently no research examining the impact of transfer effects from mental training during the glide phase on pilots’ flight performance. Accordingly, we combined EEG power spectral density, sample entropy, and functional connectivity analyses to characterize both local neural activity and inter-regional interactions, elucidating the mechanisms through which mental training modulates cognitive-motor performance during the glide phase of the carrier-based aircraft landing and providing an evidence-based training protocol for this critical flight segment.

## 2 Materials and methods

### 2.1 Participants

Using G. Power, the sample size for this study was estimated to be 40 participants, based on effect sizes from previous relevant research (effect size dz = 0.25, *α* err prob = 0.05, 1-*β* err prob = 0.95). A total of 46 healthy volunteers were recruited and randomly assigned to either a training group or a control group. The final sample had a mean age of 22.58 ± 3.44 years (range: 18–30 years).

To ensure the reliability and consistency of the experiment, the following inclusion criteria were applied:

1. Male, right-handed, bachelor’s degree or higher;
2. Uncorrected or corrected visual acuity of 1.0 or above, with good general health;
3. No prior participation in similar simulated carrier-based flight experiments;
4. Adequate sleep the night before the experiment; and no intake of any medication affecting the central nervous system for three days prior to the experiment.

All participants provided written informed consent after being informed of the experimental procedures and potential benefits. The study protocol was approved by the medical ethics committee of Fourth Military Medical University. Participants were informed that they could withdraw from the experiment at any time without any penalty or consequence.

### 2.2 Simulated carrier-based aircraft approach and landing platform

**Figure 1:**
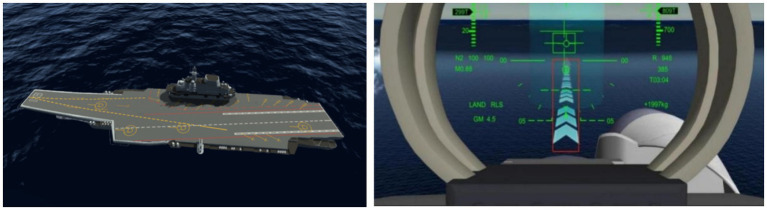
The operating interface of simulated carrier-based aircraft approach and landing platform.

The simulated carrier-based aircraft approach and landing platform was independently developed by our team. It primarily comprises a maritime visual simulation system, control system hardware, and system management software, enabling the simulation of approach and landing operations as well as escape and go-around manoeuvres under varying meteorological conditions. The operating interface is shown in Figure 1.

**Figure 2:**
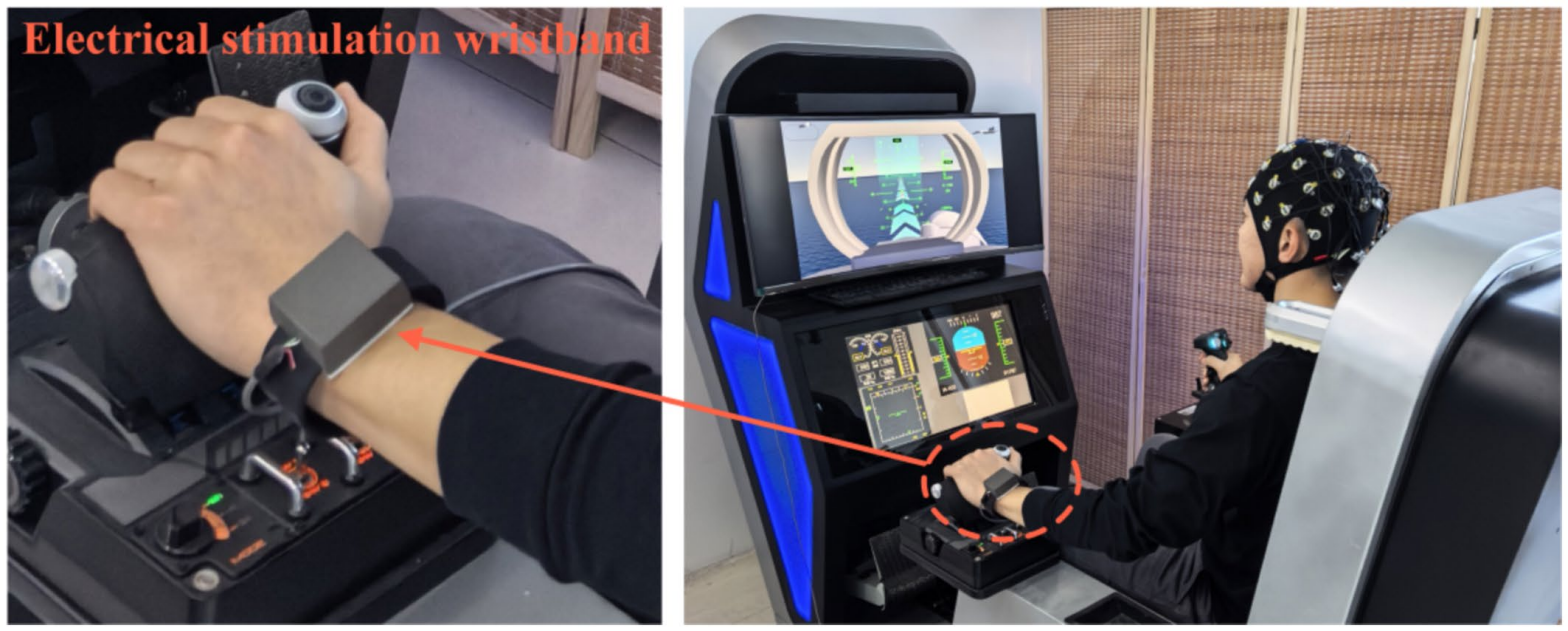
The system control platform and electrical stimulation wristband.

To align with the high-stress characteristics experienced by carrier-based aircraft pilots during approach and landing, we incorporated electrical stimulation within safe parameters as a stressor in our experimental task design. This simulates the psychophysiological stress state encountered by carrier-based aircraft pilots during approach and landing. To ensure absolute participant safety while maintaining the ability to induce a psychophysiological stress response, we adopted a conservative parameter set. Electrical stimulation was delivered as trains of biphasic pulses with a frequency of 50 Hz and a pulse width of 200 μs. Each stimulus train lasted 300 ms and was delivered at 10-s intervals during the formal task. Intensity was individually calibrated based on the sensory threshold (mean = 6.8 mA) and set to 2–3 times this value, with a strict upper limit of 20 mA (resulting mean intensity = 14.5 mA). The system control platform and electrical stimulation wristband are depicted in Figure 2.

### 2.3 Mental training system

**Figure 3:**
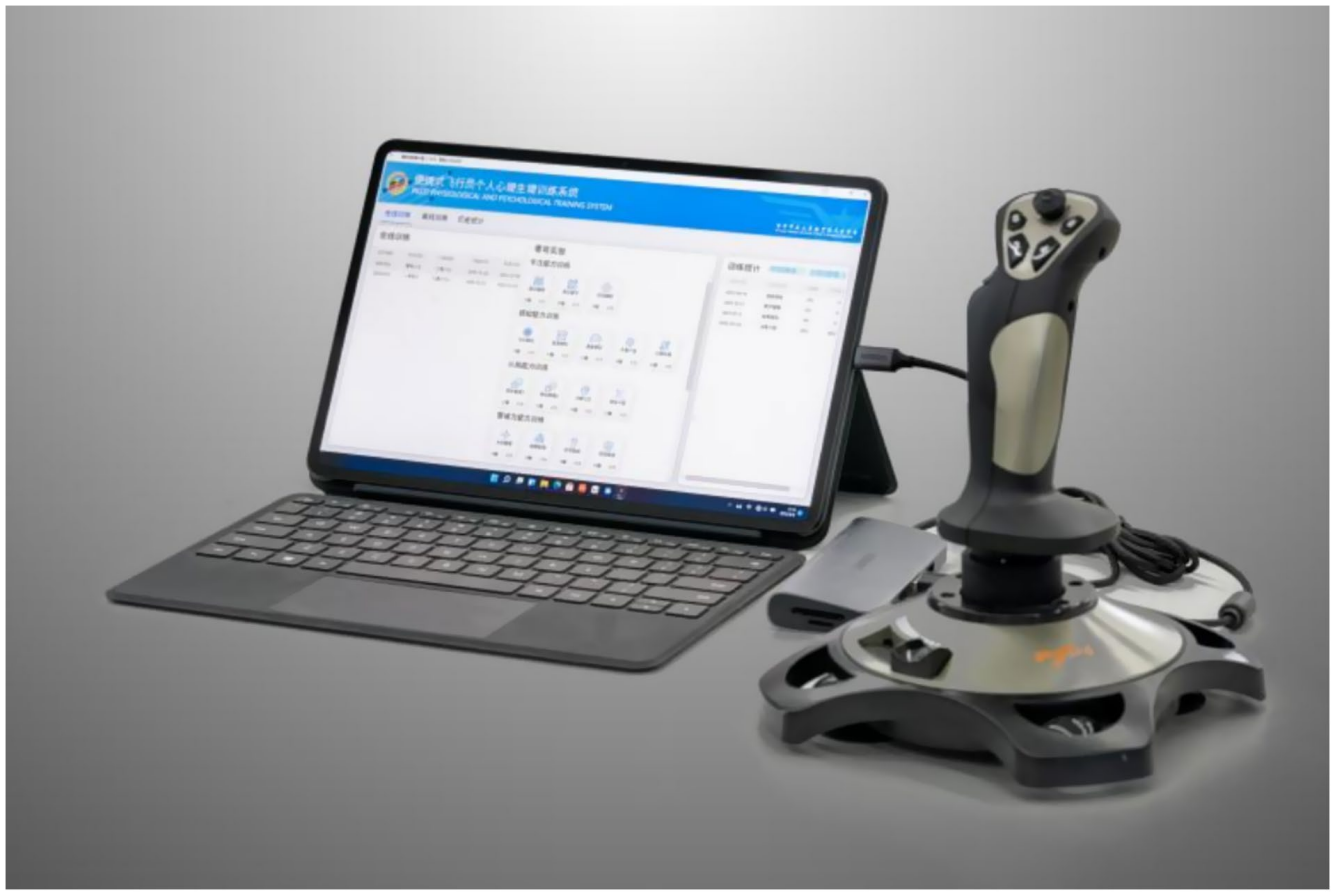
The portable pilot mental training system.

The mental training was conducted using a portable pilot mental training system developed by our team, as illustrated in Figure 3. The training program comprised six modules: multi-task training, three-dimensional trajectory training, distance perception training, orientation perception training, velocity perception training, and attention-memory training.

The multi-task training module was designed to enhance the ability to integrate global information elements. Its interface presented four types of task-related information that participants were required to extract, assess, and integrate via the visual system to elicit appropriate responses. The module consisted of three primary tasks (a light-point task, a pursuit task, and an instrument task) and one secondary task (a digital reaction task), with a total duration of 5 min. When the primary and secondary tasks were presented simultaneously, participants were instructed to prioritize the primary tasks and to attend to the secondary task only when residual attentional capacity permitted. The light-point task simulated discrete burst-type operations: between 11 and 18 randomly positioned dots appeared on the interface, and participants, after counting the dots, responded by pressing the fire button on the control stick followed by the corresponding number key. The instrument task simulated sustained monitoring and discrete control operations: a rotating pointer moved continuously across a dial, and participants were required to press the A button on the control stick together with the corresponding dial number key whenever the pointer entered a red zone. The pursuit task simulated continuous tracking control operations: participants manipulated a control stick to keep a reticle aligned with a moving target; an audible alert was automatically triggered if the target escaped the reticle, prompting timely correction. The digital reaction task, presented on the right side of the screen, required participants, only when primary tasks did not fully occupy attentional resources, to hold down the B button on the control stick while pressing the number key corresponding to the digit displayed. The objective of this module was to develop the capacity to integrate head-up display (HUD) information with spatial scene information relevant to landing operations.

The three-dimensional trajectory training module aimed to improve upper limb goal-oriented motor skills by exercising the hand-eye-brain control loop. Participants manipulated a joystick to keep a ball rolling smoothly along a track within a three-dimensional virtual environment. The track featured varying degrees of curvature and gradient. The joystick direction determined the ball’s heading, and the deflection distance governed its velocity. Participants were required to guide the ball from a starting point to a destination along the track as smoothly and rapidly as possible. Each successful arrival at the destination constituted one completed trial. The total experimental session lasted 360 s, and performance was determined by both the number of successfully completed trials and the total distance traversed by the ball.

The distance perception training module exercised participants’ spatial working memory for distance perception. At the beginning of the training session, several square grids of sizes 5 × 5, 7 × 7, and 10 × 10 were presented sequentially. Each grid contained a pair of points with distinct inter-point distances. Participants were required to click on the grids in ascending order according to the distance between the two points within each grid. A correct selection turned the border of the grid green, whereas an incorrect selection turned the border red, requiring the participant to continue selecting until all the grids had been properly ordered.

The orientation perception training module enhanced spatial working memory for orientation perception. At the beginning of the training session, a configuration of 11 concentric circles was presented. The innermost circle had a radius of 100 units from the center, with each subsequent circle spaced at intervals of 100 units. In addition, 12 radial lines extended outward from the center, with an angular interval of 15 degrees between adjacent lines. The ray pointing directly upward was designated as 0 degrees, the ray pointing to the right as 90 degrees, the downward ray as 180 degrees, and the leftward ray as 270 degrees. These radial lines and concentric circles partitioned the display into multiple distinct sectors. Participants were required to click on the sector corresponding to the coordinates presented in the upper right corner of the screen. Correct or incorrect responses were followed by immediate feedback shown on the right side of the interface.

The velocity perception training module improved spatial working memory for velocity perception. After the training session began, the flight speed and initial velocity were provided. Upon entering the cloud, participants were required to estimate the time taken to pass through the cloud, and their response was recorded as the time of keyboard press. The test results displayed the actual input time, the actual time of the aircraft passing through the cloud, and the time difference between the two. Each test consisted of 10 trials.

These three modules (distance, orientation, and velocity perception) were designed to train participants in perceiving the position, orientation, and relative velocity of the aircraft carrier during landing operations.

The attention-memory training module exercised participants’ working memory capacity through repeated memorization exercises involving patterns within a nine-square grid. The module consisted of 10 trials. In each trial, a set of images arranged in a nine-square grid was first presented on the screen for 2 s before disappearing. Subsequently, a blank nine-square grid was displayed, with a question mark appearing at a randomly selected location. Participants were required to select, from the images presented below the grid, the image that had originally appeared at the marked location based on their memory.

**Figure 4:**
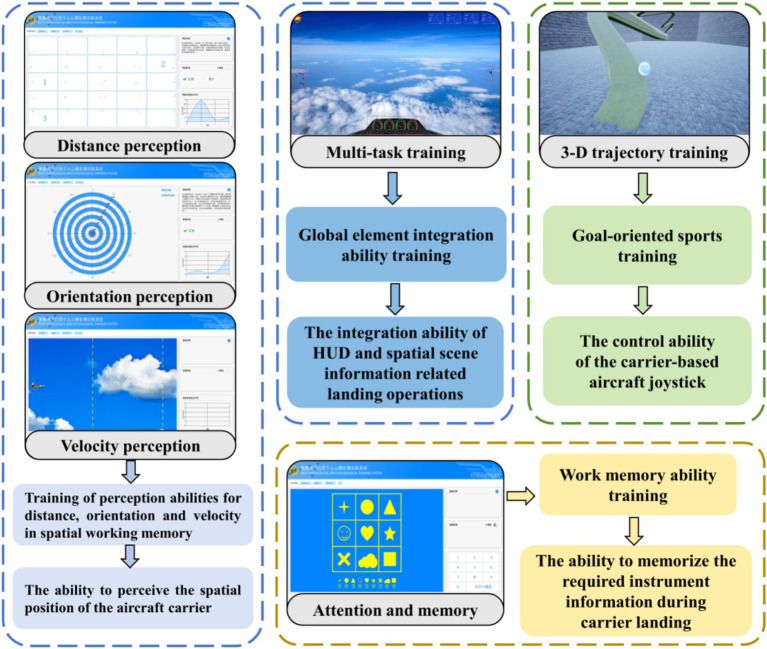
The capability enhancement modules corresponding to the mental training.

Figure 4 illustrates the operational interface and the corresponding capability enhancement modules for each component of the mental training paradigm.

### 2.4 EEG equipment

EEG signals were recorded using a 32-channel wireless portable EEG system with a LiveAmp amplifier (Brain Products GmbH, Gilching, Germany). The signals were transmitted via fibre-optic technology over a range of 10 metres, enabling accurate reproduction of faint brainwave signals.

**Figure 5:**
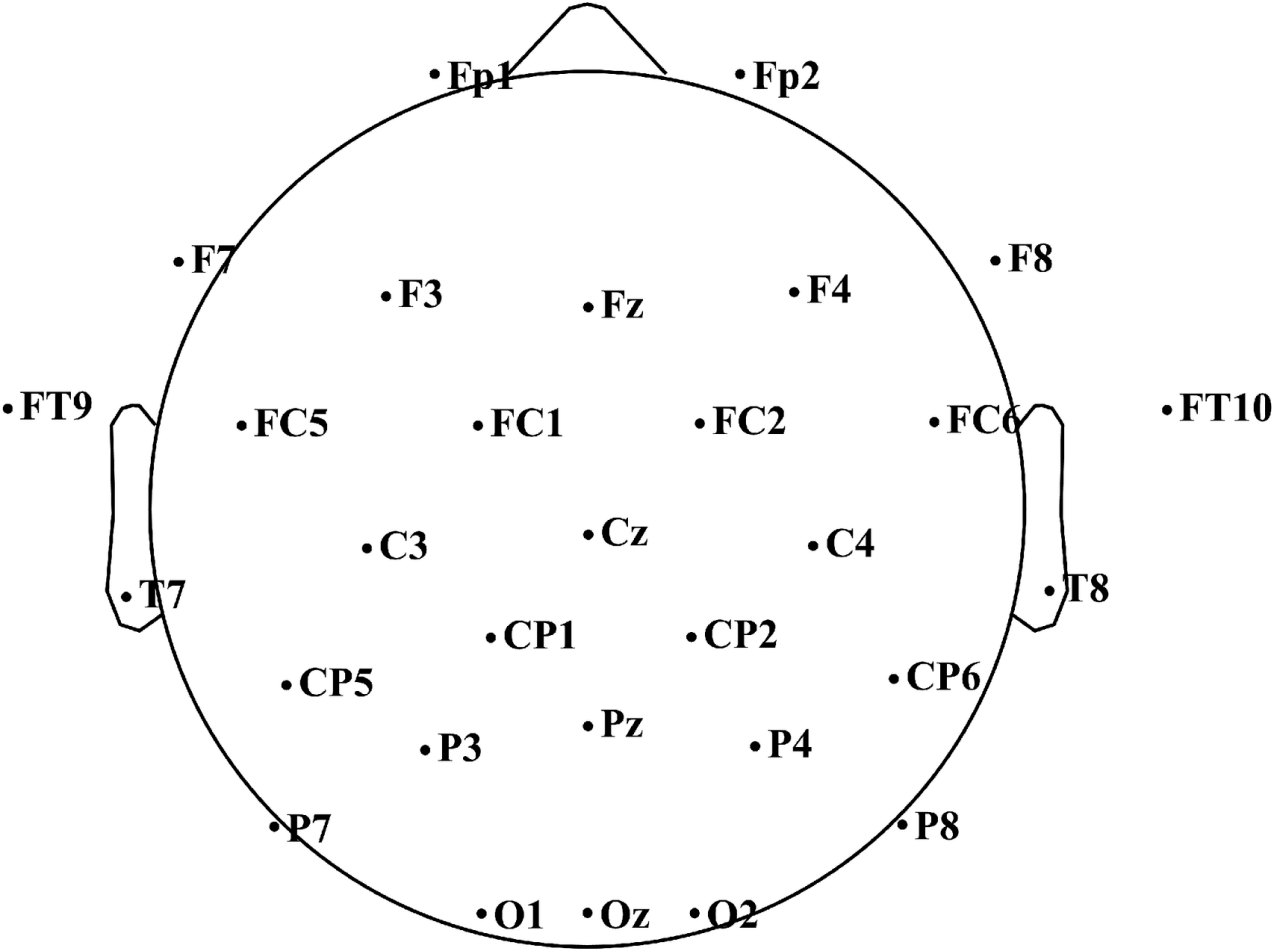
The positions of electrodes.

EEG was recorded using 32 passive Ag/AgCl ring electrodes mounted in an elastic cap according to the extended international 10–20 system. The electrode sites included: Fp1, Fp2, Fz, Ft9, Ft10, F3, F4, F7, F8, Fc1, Fc2, Fc5, Fc6, C3, C4, Cz, CP1, CP2, CP5, CP6, Pz, P3, P4, P7, P8, O1, O2, Oz, T7, and T8, as illustrated in Figure 5. The ground electrode was placed between Fp1 and Fp2, and the reference electrodes were placed at the bilateral mastoids (TP9 and TP10). A conductive gel was applied to ensure stable skin-electrode contact, and electrode impedances were maintained below 5 kΩ throughout the recording session. The sampling rate was set to 250 Hz.

Furthermore, this experiment was conducted in a basement to minimize external environmental influences on the accuracy of the EEG data, with overall noise levels maintained below 20 dB. Uniform and stable white light illumination was employed throughout to prevent external light sources from affecting the experimental results.

### 2.5 Experimental steps

**Figure 6:**
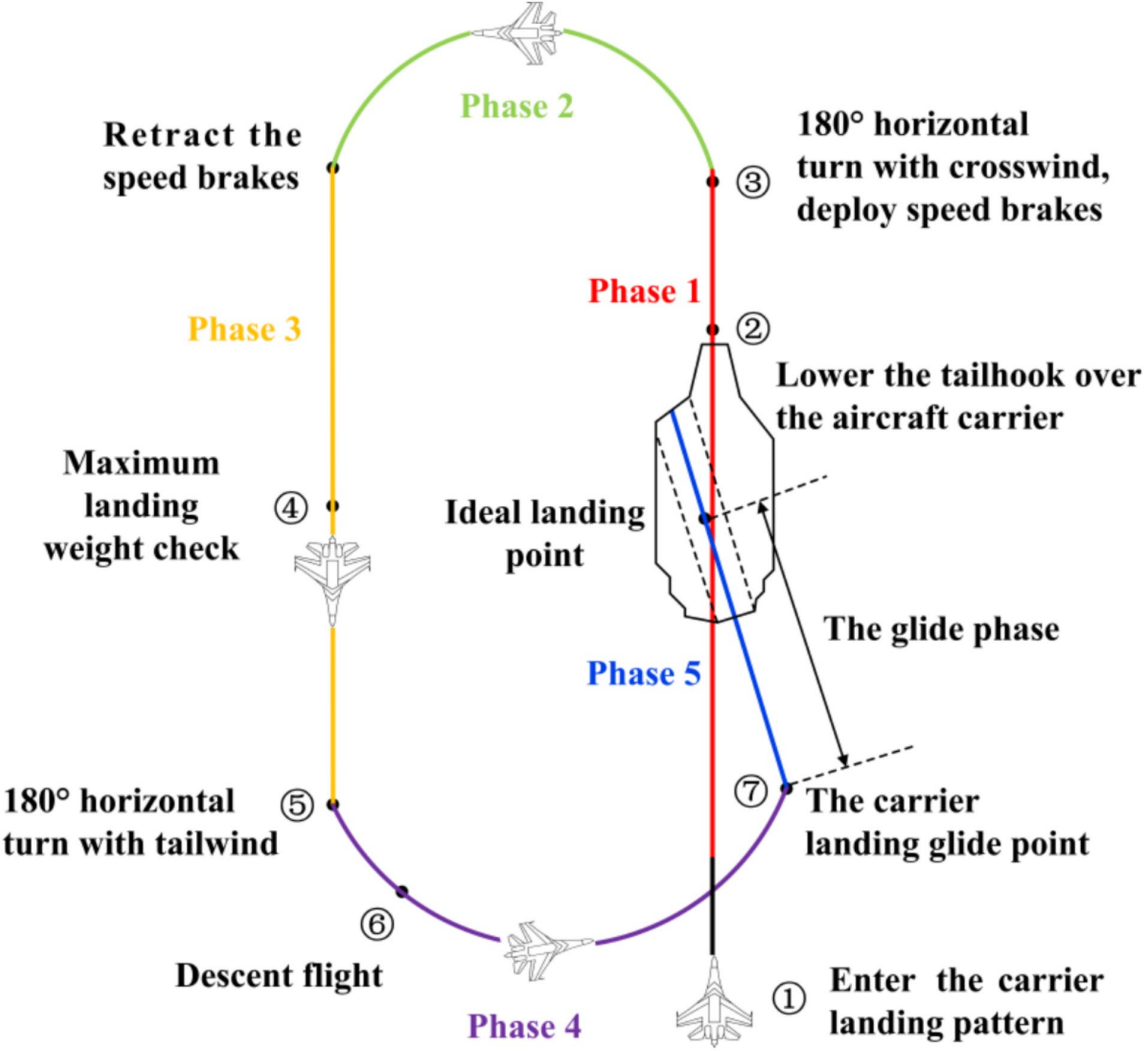
The flight path of the carrier-based aircraft during landing.

Each participant underwent a 10-day flight simulation training course, consisting of one hour of training per day. Training strictly adhered to the maneuver requirements for approach and landing, minimizing the impact on the experiment caused by participants’ unfamiliarity with the flight simulation tasks. Training course includes basic instrument observation, control stick and throttle lever operation techniques, flight trajectories, and key flight operation principles. The entire carrier landing process begins above the aircraft carrier, proceeds around the carrier to the landing glide point at its stern, and concludes with the aircraft gliding down the arresting gear to land. The flight path is illustrated in Figure 6. The operational procedures for the simulated carrier landing approach task are as follows: the participant manipulates the aircraft’s heading via the control stick and rudder pedals, while the throttle lever regulates airspeed, following the completion of the airfield traffic pattern, the aircraft is successfully landed on the carrier deck. Upon the appearance of the “Decelerate and turn for preparation” prompt during the flight task, participants were instructed to reduce airspeed and execute the turn by reference to the guidance lines and instruments. The maneuver was performed in accordance with the voice instructions provided throughout the flight.

Before proceeding to the formal test, each participant completed repeated practice sessions for the simulated landing task to master the operational procedures. During formal testing, the experimenter used standardized instructions to ensure that participants fully understood the testing process. Each participant first completed a practice session to thoroughly recall the key flight techniques and precautions from the flight simulation course. Testing commenced once participants were fully prepared. Prior to the flight experiment, participants donned the EEG cap with the experimenter’s assistance, adjusted the EEG electrodes, and secured the electrical stimulation wristband on their right wrist.

First, flight data and EEG data were collected from participants prior to mental training. Each participant’s formal testing session lasted approximately 10 min, comprising about 5 min for familiarization with the operating environment and about 5 min for the experimental procedure. Each participant completed only one dynamic carrier-based landing task per testing session, as each full landing approach lasted approximately 3–4 min. This design was chosen to minimize fatigue, which could otherwise confound both EEG and performance data, and to preserve ecological validity by mirroring the demands of a single real-world landing mission. While this approach precluded trial averaging, it prioritized participant comfort and task authenticity. Upon task completion, the computer automatically stored flight data, key point labeling data, and EEG data into the database.

**Figure 7:**
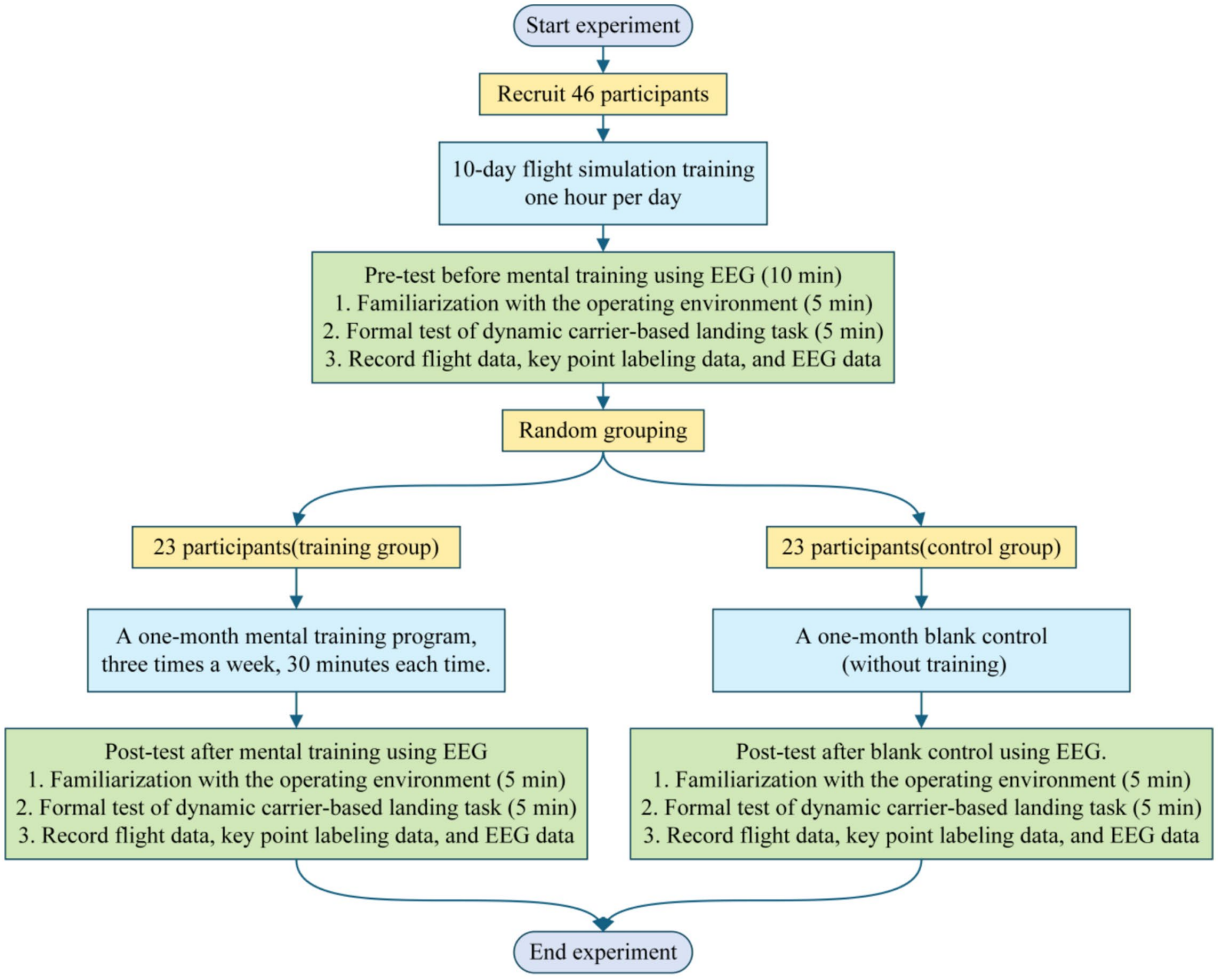
The general flowchart of the experiment.

Second, the 46 participants were randomly assigned to two groups: a training group and a control group, with 23 participants in each group. The training group underwent one month of mental training, consisting of three sessions per week, each lasting approximately 30 min. The training content included multi-task training, three-dimensional trajectory training, distance perception training, orientation perception training, velocity perception training, and attention-memory training. One month later, participants in the training group underwent another simulated landing task test, with flight data, key point labeling data, and EEG data collected and stored in the database. The control group received no training and served as a blank control for one month. One month later, they again performed simulated landing tests, with data collected and stored in the database. The general flowchart of the experiment is as shown in Figure 7.

### 2.6 Data processing

During the carrier landing approach, pilots are required to maintain a steady descent angle between 8 and 11 degrees to ensure a successful touchdown. Both flight time and glide path stability are critical factors for mission execution. To comprehensively evaluate flight performance, a composite indicator F was derived as follows:

Let D denote the standardized score of the sum of absolute deviations from the standard glide path, and T denote the standardized score of flight time. The flight performance evaluation indicator F was calculated as: F=(T+D)/2 .

This composite score was computed for each participant in each testing session and used for subsequent statistical analyses.

EEG data preprocessing was performed using EEGLAB running under MATLAB R2022b. The continuous EEG data were originally recorded with reference to the bilateral mastoid electrodes (TP9 and TP10). For subsequent analyses, the data were re-referenced offline to the common average reference, computed as the average signal across all 30 recording channels. The sampling rate remained at 250 Hz, as no downsampling was required for the subsequent analyses. Bad channels were identified by visual inspection of the power spectral density and time series, as well as by examining their correlation with neighboring channels. Channels exhibiting flat lines, excessive high-frequency noise, or atypical signal amplitude were marked as bad. The continuous data were then filtered using a finite impulse response (FIR) bandpass filter with cutoffs at 1 Hz and 30 Hz. This frequency range was selected because it encompasses the primary frequency bands of interest (delta, theta, alpha, beta) while effectively removing low-frequency drift and high-frequency noise.

To correct ocular and motion artifacts, independent component analysis (ICA) was performed on the filtered continuous data using the Infomax algorithm (‘runica’ in EEGLAB). Components representing ocular, cardiac, and muscle artifacts were identified based on visual inspection of topographies, time courses, and power spectra. On average, 4.3 ± 1.5 components (range: 2–7) were removed per participant. The remaining components were back-projected to obtain artifact-corrected EEG data.

From each participant’s artifact-corrected EEG recording, a continuous 180-s segment was extracted for further analysis. This segment was selected from the middle portion of the 5-min formal landing task, starting approximately 60 s after task onset and ending approximately 60 s before task completion. This ensured that the analyzed window captured stable task engagement while avoiding the initial transition period and any end-of-task artifacts. The same temporal anchoring relative to task onset was applied consistently across all participants to ensure comparability.

Power spectral density (PSD) was computed for each channel using Welch’s method implemented in the MATLAB ‘pwelch’ function. The following parameters were used: Hanning window with a window length of 500 samples (2 s), 50% overlap between consecutive windows (250-sample overlap-shift), and Fourier transform length of 500 samples, yielding a frequency resolution of 0.5 Hz. PSD was computed for the full 1–30 Hz range and subsequently averaged within the following frequency bands of interest: delta (1–3 Hz), theta (3–7 Hz), alpha (8–12 Hz), and beta (13–30 Hz). For each band, absolute power was extracted and log-transformed to approximate normality for subsequent statistical analyses.

This study employed sample entropy (SE) to characterize the complexity of EEG signals. The findings indicate that signal complexity exhibits a positive correlation with entropy values, meaning higher complexity corresponds to greater entropy. By employing the SE method, we can better assess the complexity of EEG signals, thereby revealing their intrinsic characteristics and patterns of change. The specific calculation process of the SE algorithm is as follows:

Step 1: the original signal with N sample points: X=[x(1),x(2),…,x(N)]

Step 2: form a set of m-dimensional vectors from the continuous points of the signal: U(i)=[x(i),x(i+1),…,x(i+m−1)], where, i=1,2,…,N−M .

Step 3: let the distance d[U(i),U(j)] between vectors U(i) and U(j) be the maximum difference among their corresponding elements, as shown in Equation 1.


$$d[U_{m}(i),U_{m}(j)]=\max(|x(i+k)-x(j+k)|)$$ (1)

Where, k=0,1,…,m−1 , i,j=1,2,…,N−m , i≠j ;

Step 4: set threshold r, when i≤N−m , Calculate the number Nm of instances where $d[U_{m}(i),U_{m}(j)]$ is less than r, and its ratio to the total number of distances N-m-1, as shown in Equation 2.


$$C_{m}(i)=N_{m}/(N-m-1)$$ (2)

The average over all i was calculated. Equation 3 is derived from this result.


$$C_{m}=[\sum_{i=1}^{N-m}C_{m}(i)]/(N-m)$$ (3)

Step 5: increase the dimension by m + 1, repeat the above step1-4, calculate $C_{m+1}$ .

Theoretically, the SE of this sequence is given by Equation 4.


$$\mathrm{SE}(m,r)=\lim_{N\to \infty }\{-\ln(C_{m+1}/C_{m})\}$$ (4)

When N is finite, the approximate value of SE is given by Equation 5.


$$\mathrm{SE}(m,r)=-\ln(C_{m+1}/C_{m})$$ (5)

SE is a method for measuring similarity between vectors, reflecting the probability that high-dimensional vectors remain similar when an additional dimension is added. Since SE does not consider comparisons between a vector and itself, its value can be precisely calculated by computing the negative average natural logarithm of the conditional probability. This calculation method is independent of vector length, making it insensitive to variations in data length and less affected by data loss, thereby exhibiting excellent noise resistance.

Due to the presence of the brain volume conductor, electrical signals from adjacent channels may exhibit spurious connectivity. The phase lag index (PLI) is highly insensitive to volume conduction effects. PLI measures the asymmetry in the distribution of phase differences between two signals, reflecting whether the phase of one signal is consistently ahead of or behind that of the other. This property makes it well suited for assessing phase synchronization between different event-related potential (ERP) channels. Therefore, in this study, PLI was employed to construct functional connectivity networks.

For any two EEG signals $x_{j}(t)$ and $x_{k}(t)$ , the phase difference between them at time t can be expressed as:


$$|\Delta \phi_{n,m}(t)|=|n\phi_{j}(t)-m\phi_{k}(t)|<\text{const}$$ (6)

Where n and m are integers. In neuroscience applications, n = m = 1 is typically chosen, with $\phi_{j}$ and $\phi_{k}$ representing the instantaneous phases of the respective time series. If the two time series exhibit synchronous fluctuations, the phase difference in Equation 6 approaches a constant value. The instantaneous phase of a time series $x_{j}(t)$ is given by Equation 7.


$$\phi_{j}(t)=\arctan\frac{{\tilde{x}}_{j}(t)}{x_{j}(t)}$$ (7)

Where ${\tilde{x}}_{j}(t)$ denotes the Hilbert transform of $x_{j}(t)$ , which is given by Equation 8.


$${\tilde{x}}_{j}(t)=\frac{1}{\pi }\mathrm{PV}\int_{-\infty }^{\infty }\frac{x_{j}(\xi )}{t-\xi }\mathrm{d\xi}$$ (8)

Here, *PV* represents the Cauchy principal value. For two time series of length *M*, the *PLI* is defined as a measure of the asymmetry in the distribution of phase differences, which is given by Equation 9.


$$\mathrm{PLI}=|\frac{1}{M}\sum_{t=0}^{M-1}\mathrm{sign}(\Delta \phi (t_{t}))|,0\leq \mathrm{PLI}\leq 1$$ (9)

Where *sign* denotes the signum function. The asymmetry refers to the unequal probabilities of the phase difference Δϕ falling within the intervals (−π,0) and (0,π) . Such asymmetry indicates that a non-zero phase difference always exists between the two time series.

### 2.7 Statistical analysis

Statistical analyses were performed using SPSS version 24.0. Descriptive statistics were calculated for all measures and are presented as mean±standard deviation. The normality of data distribution was assessed using the Shapiro–Wilk test.

For flight performance data that violated the normality assumption, non-parametric tests were employed: the Wilcoxon signed-rank test for within-group comparisons (pre-test versus post-test) and the Mann–Whitney U test for between-group comparisons (between the training and control groups at post-test).

For EEG data that satisfied the normality assumption, within-group comparisons were conducted using paired-sample t-tests, as our primary focus was to examine improvements within each group following the intervention.

To examine the relationship between brain functional changes and flight performance, Pearson correlation coefficients were calculated between EEG-derived indicators (power spectral density and functional connectivity values) and flight performance metrics (glide path deviation D, flight time T, and the composite indicator F). For EEG indicators, we selected electrodes and frequency bands that showed significant changes after mental training in the training group.

To control for multiple comparisons, the false discovery rate (FDR) method was applied, and statistical significance was set at *p* < 0.05.

## 3 Results

### 3.1 Comparison of carrier landing approach performance before and after mental training

Flight performance was evaluated using a composite indicator F, derived from standardized scores of glide path deviation and flight time. For the 23 participants in the training group, flight performance improved significantly from pre-test to post-test (Z = −2.859, *p* = 0.004), whereas no significant change was observed for the 23 participants in the control group (Z = −0.395, *p* = 0.693).

Between-group comparisons revealed that after mental training, the training group demonstrated significantly better performance than the control group on both glide path deviation (U = 167.000, Z = −2.143, *p* = 0.032) and flight time (U = 72.000, Z = −4.229, *p* = 0.000).

### 3.2 Comparison of EEG PSD results before and after mental training

**Figure 8:**
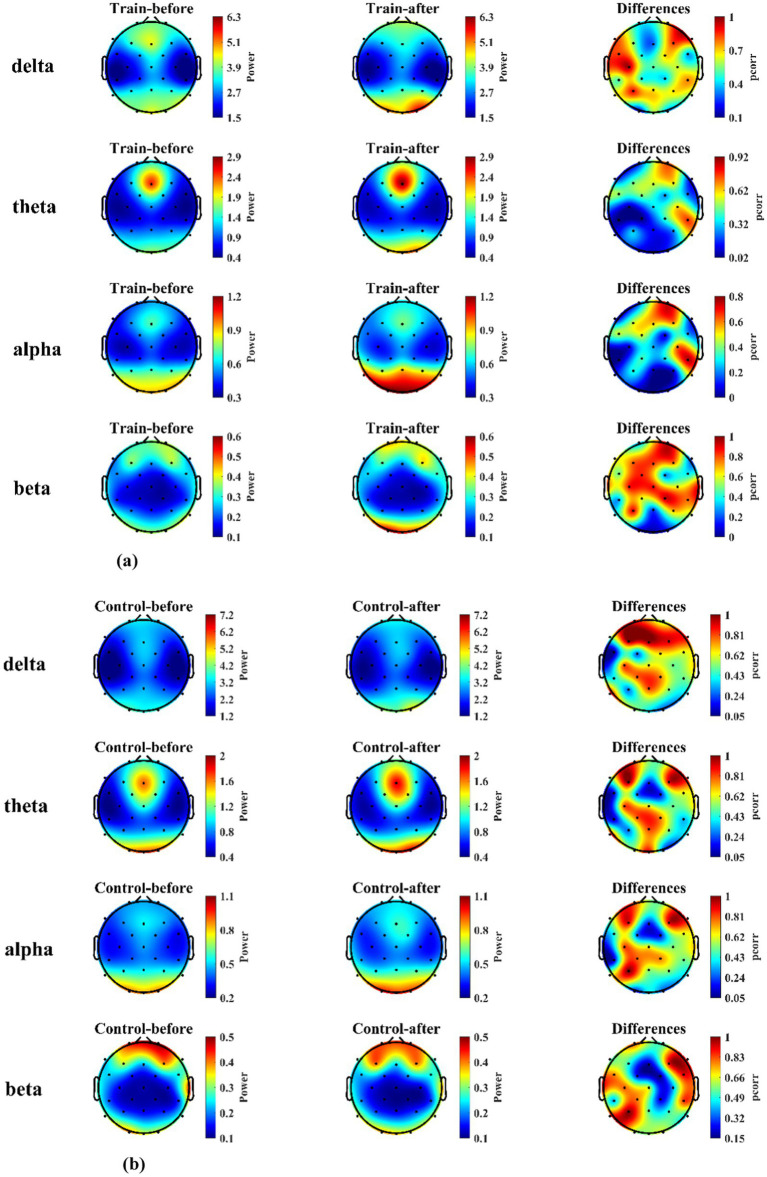
The brain maps of PSD and statistical differences between training and control groups before and after mental training. **(a)** PSD levels and statistical differences in the training group (*n* = 23). **(b)** PSD levels and statistical differences in the control group (*n* = 23).

For the training group, EEG power spectral density (PSD) analysis during the landing task showed that compared to pre-training, PSD in the alpha band at electrodes F7, CP5, Pz, O1, and Oz, in the theta band at electrodes C3, CP5, and CP1, and in the beta band at electrodes O1 and Oz was significantly increased after training (*p* < 0.05). The electrodes showing significant differences and their corresponding statistical values are listed in Table 1. In the control group, no significant differences in PSD were observed across all frequency bands and electrodes after the blank control (*p* > 0.05). The distribution of PSD values across electrodes for both groups, along with their differences, is presented in Figures 8a,b.

**Table 1:** The electrodes showing significant differences in PSD between before and after mental training of training group (*n* = 23).

Electrode	Frequency band	$\bar{X}$ ± S	t (22)	*p*
F7	alpha	0.40 ± 0.23 (before mental training)	−2.84	0.038^*^
		0.52 ± 0.30 (after mental training)		
C3	theta	0.51 ± 0.23 (before mental training)	−2.83	0.039^*^
		0.61 ± 0.32 (after mental training)		
CP5	theta	0.58 ± 0.30 (before mental training)	−2.78	0.022^*^
		0.70 ± 0.33 (after mental training)		
CP5	alpha	0.46 ± 0.34 (before mental training)	−5.00	0.000^*^
		0.63 ± 0.42 (after mental training)		
CP1	theta	0.62 ± 0.26 (before mental training)	−3.08	0.022^*^
		0.72 ± 0.29 (after mental training)		
Pz	alpha	0.69 ± 0.44 (before mental training)	−2.82	0.040^*^
		0.91 ± 0.69 (after mental training)		
O1	alpha	0.92 ± 0.41 (before mental training)	−2.71	0.025^*^
		1.26 ± 0.59 (after mental training)		
O1	beta	0.38 ± 0.23 (before mental training)	−2.78	0.025^*^
		0.59 ± 0.35 (after mental training)		
Oz	alpha	0.92 ± 0.39 (before mental training)	−3.24	0.015^*^
		1.23 ± 0.49 (after mental training)		
Oz	beta	0.37 ± 0.22 (before mental training)	−2.81	0.020^*^
		0.54 ± 0.34 (after mental training)		

*p < 0.05.

### 3.3 Comparison of sample entropy results before and after mental training

Sample entropy analysis for the training group revealed that compared to pre-training, sample entropy was significantly increased after training in the alpha band at electrode Pz and in the theta band at electrodes Fp1, P7, CP2, and C4, while it was significantly decreased in the alpha band at electrode Cz and in the beta band at electrode Pz (*p* < 0.05). The electrodes showing significant differences and their corresponding statistical values are listed in Table 2.

**Table 2:** Electrodes with significant differences in sample entropy across different frequency bands in the training group.

Electrode	Frequency band	$\bar{X}$ ± S (before training)	$\bar{X}$ ± S (after training)	t (22)	*p*
Fp1	theta	0.24 ± 0.01	0.25 ± 0.01	−2.99	0.027^*^
Pz	alpha	0.43 ± 0.01	0.44 ± 0.01	−2.44	0.047^*^
Pz	beta	0.58 ± 0.01	0.58 ± 0.01	2.68	0.047^*^
P7	theta	0.24 ± 0.01	0.25 ± 0.01	−3.05	0.023^*^
CP2	theta	0.24 ± 0.01	0.24 ± 0.01	−3.29	0.013^*^
Cz	alpha	0.44 ± 0.01	0.43 ± 0.01	2.72	0.049^*^
C4	theta	0.24 ± 0.01	0.25 ± 0.01	−3.36	0.011^*^

*p < 0.05.

**Figure 9:**
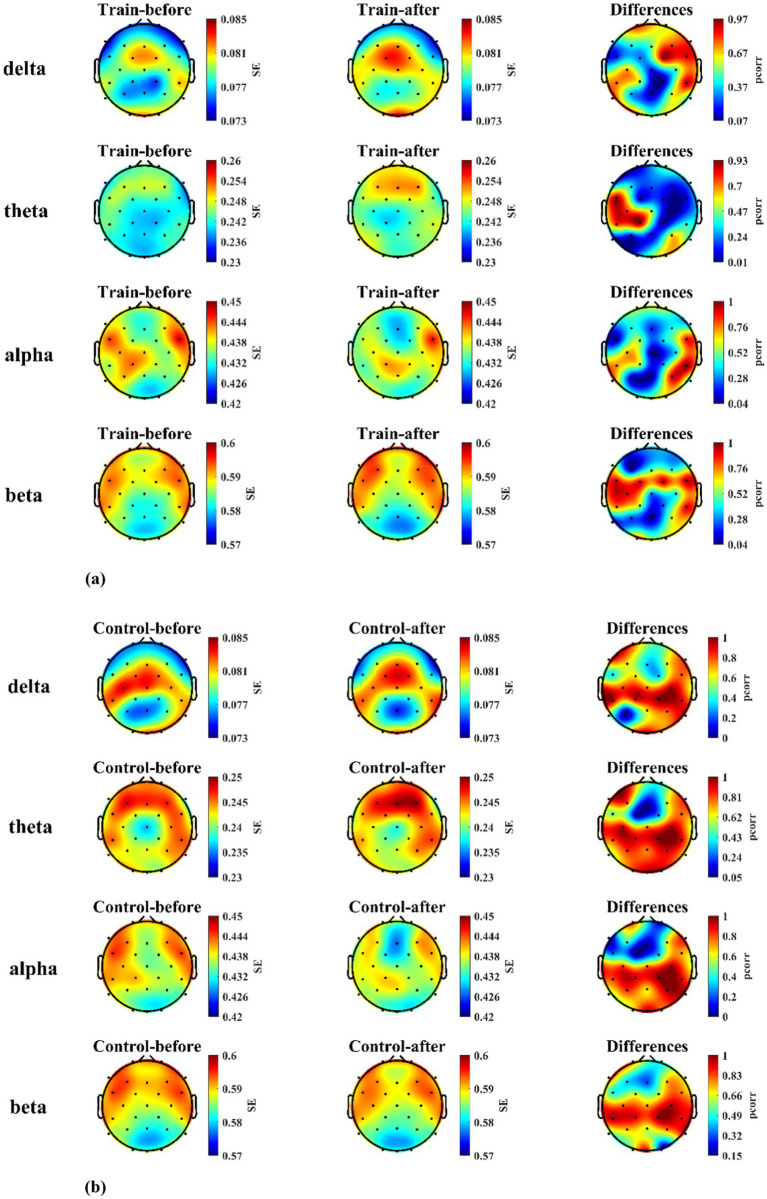
The brain maps of sample entropy and statistical differences between training and control groups before and after mental training. **(a)** Sample entropy levels and statistical differences in the training group (*n* = 23). **(b)** Sample entropy levels and statistical differences in the training group (*n* = 23).

For the control group, sample entropy analysis showed that compared to pre-control, sample entropy was significantly decreased after the blank control in the alpha band at electrodes Pz, F7, and FC1, and significantly increased in the delta band at electrode P3 (*p* < 0.05). The electrodes showing significant differences and their corresponding statistical values are listed in Table 3. Figures 9a,b display the sample entropy values for the training and control groups before and after mental training, along with the results of within-group statistical comparisons.

**Table 3:** Electrodes with significant differences in sample entropy before and after the blank control in the control group.

Electrode	Frequency band	$\bar{X}$ ± S (before training)	$\bar{X}$ ± S (after training)	t (22)	*p*
Pz	alpha	0.44 ± 0.01	0.43 ± 0.01	2.76	0.046^*^
F7	alpha	0.44 ± 0.01	0.44 ± 0.01	3.13	0.019^*^
FC1	alpha	0.44 ± 0.01	0.43 ± 0.01	3.98	0.002^*^
P3	delta	0.08 ± 0.01	0.08 ± 0.00	−3.22	0.016^*^

*p < 0.05.

### 3.4 Comparison of functional connectivity results before and after mental training

**Figure 10:**
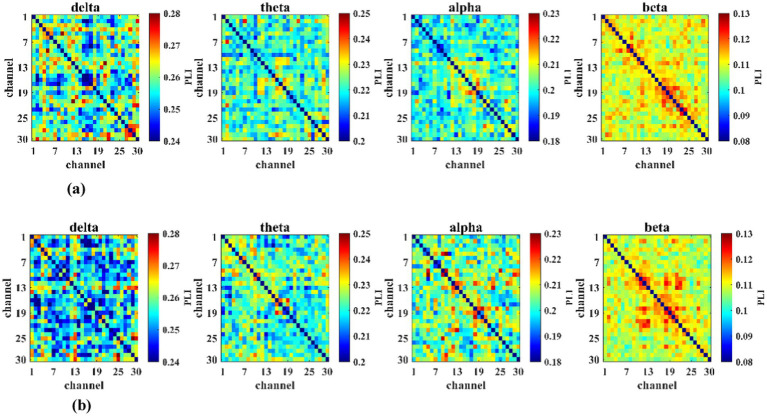
Brain functional connectivity matrices across frequency bands before and after mental training in the training group (*n* = 23). **(a)** Brain functional connectivity matrix diagram before mental training in the training group. **(b)** Brain functional connectivity matrix diagram after mental training in the training group.

**Figure 11:**
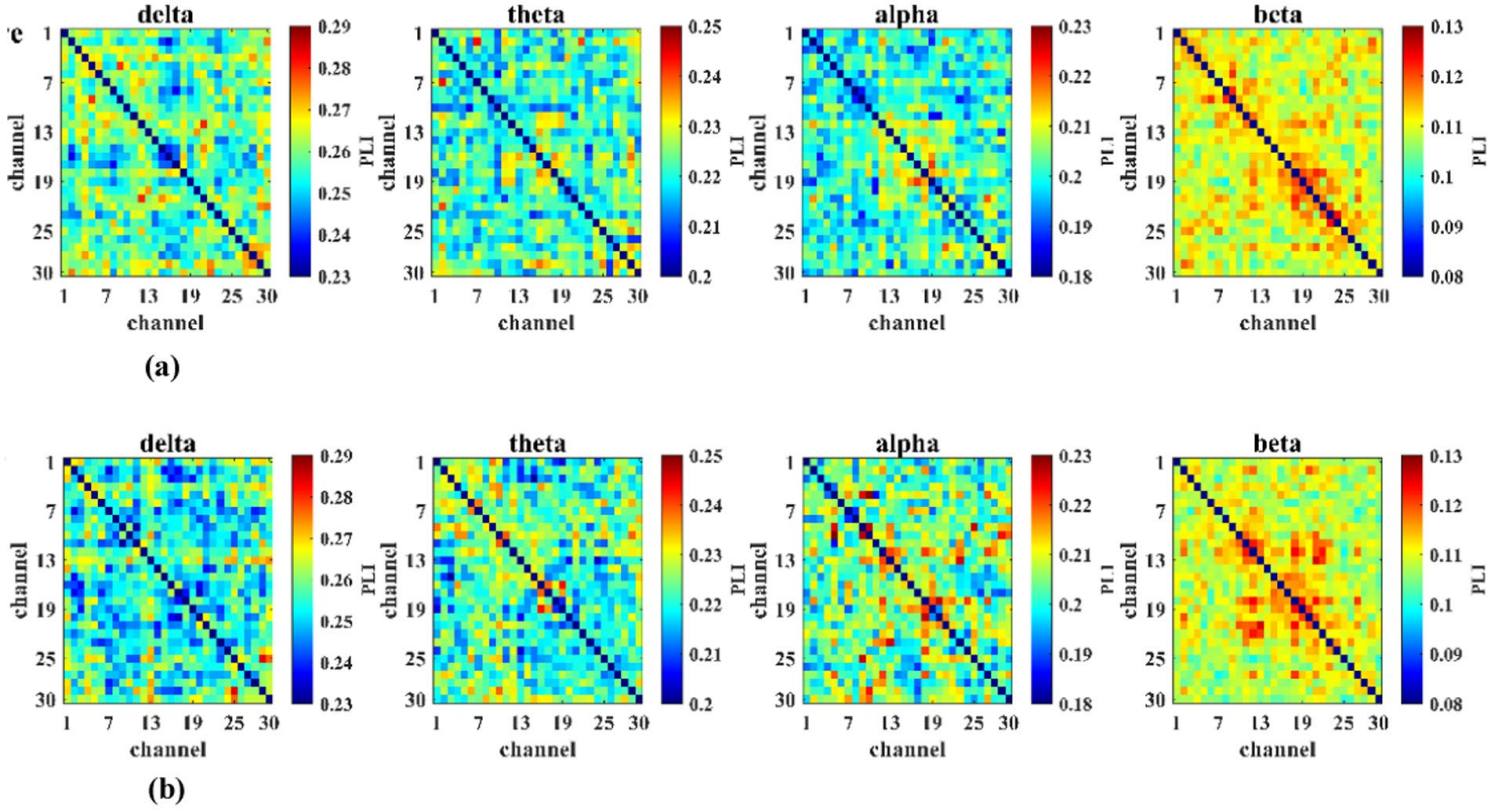
Brain functional connectivity matrices across frequency bands before and after mental training in the control group (*n* = 23): **(a)** Brain functional connectivity matrix diagram before mental training in the control group; **(b)** brain functional connectivity matrix diagram after mental training in the training group.

**Figure 12:**
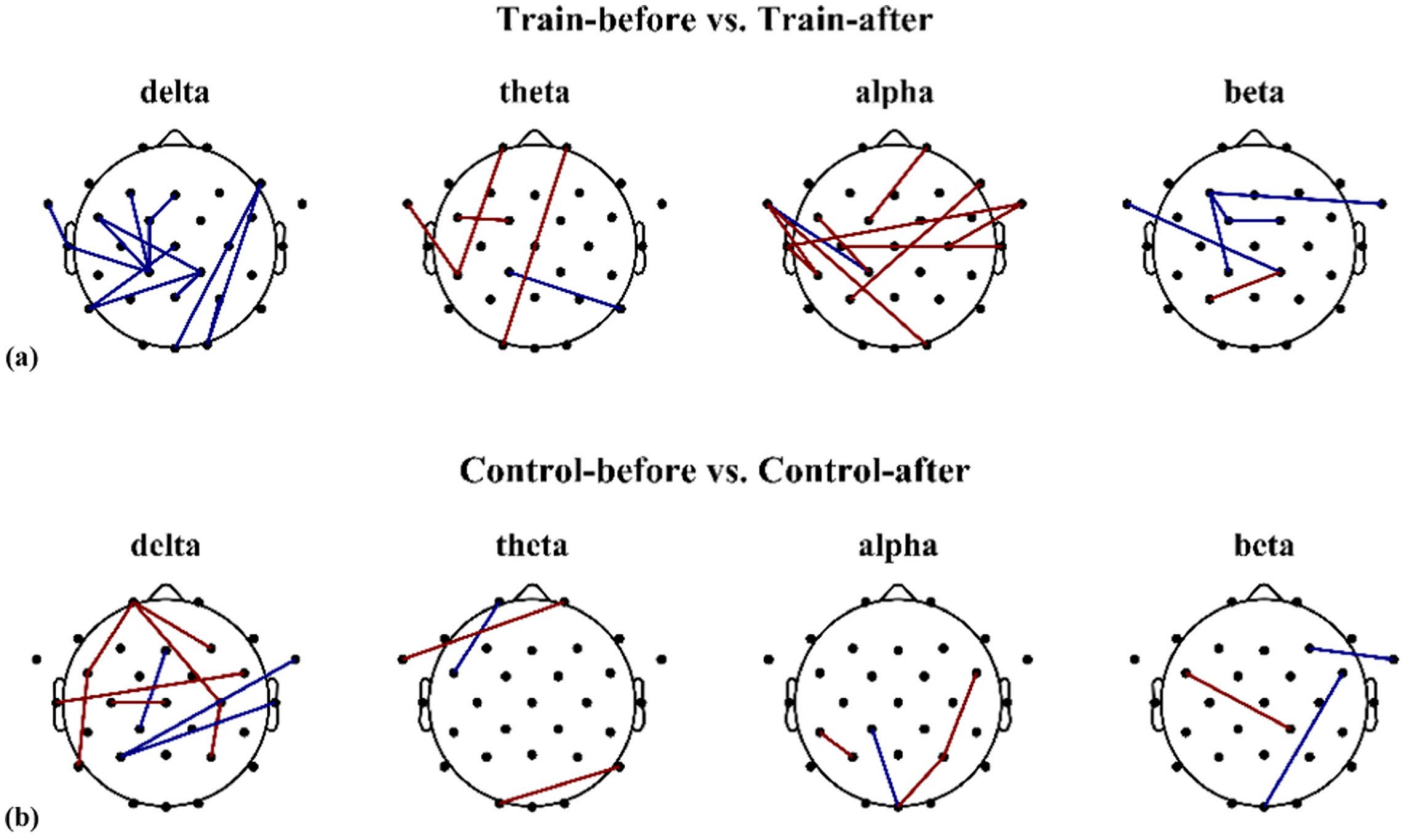
Schematic diagram of different electrodes showing functional connectivity differences between the training group and control group before and after training. **(a)** Schematic diagram of electrodes showing differences in brain functional connectivity before and after training in the training group (*n* = 23); **(b)** schematic diagram of electrodes showing differences in brain functional connectivity before and after training in the control group (*n* = 23).

The functional connectivity matrices for the delta, theta, alpha, and beta frequency bands in the training group and control group before and after mental training are shown in Figures 10a,b, 11a,b, respectively. Schematic diagrams of functional connectivity differences between the two groups before and after intervention are presented in Figures 12a,b. Blue lines indicate significant differences (*p* < 0.05) in functional connectivity between electrode pairs, with connectivity strength greater before than after training; red lines indicate significant differences (*p* < 0.05), with connectivity strength greater after training than before.

For the training group, significant differences in functional connectivity before and after mental training were observed in the following electrode pairs: delta band: Fz-FC1, FT9-T7, F3-CP1, FC5-CP1, FC1-CP1, T7-CP1, O2-P4, FC5-CP2, Pz-CP2, P7-CP2, P7-Cz, Oz-F8, O2-F8; theta band: FC5-FC1, Fp1-CP5, FT9-CP5, CP1-P8, O1-Fp2; alpha band: FT9-CP5, T7-CP5, FT9-CP1, FC5-CP1, FT9-O2, C3-T8, T7-FT10, C4-FT10, P3-F8, FC1-Fp2; beta band: F3-FC1, F3-CP1, FT9-CP2, P3-CP2, F3-FT10, FC1-FC2. Detailed statistical results for these electrode pairs are presented in Table 4.

**Table 4:** Electrodes with significant differences in functional connectivity before and after training in the training group (*n* = 23, $\bar{X}$ ± S).

Connection between electrodes	Frequency band	Before training	After training	t (22)	*p*
Fz-FC1	delta	0.27 ± 0.03	0.24 ± 0.03	3.26	0.014^*^
FT9-T7	delta	0.28 ± 0.03	0.25 ± 0.03	2.89	0.034^*^
F3-CP1	delta	0.26 ± 0.03	0.24 ± 0.03	3.69	0.001^*^
FC5-CP1	delta	0.27 ± 0.04	0.24 ± 0.03	2.68	0.031^*^
FC1-CP1	delta	0.27 ± 0.03	0.24 ± 0.03	2.82	0.040^*^
T7-CP1	delta	0.26 ± 0.03	0.23 ± 0.02	3.34	0.012^*^
O2-P4	delta	0.27 ± 0.04	0.24 ± 0.04	3.14	0.019^*^
FC5-CP2	delta	0.27 ± 0.03	0.24 ± 0.03	2.98	0.027^*^
Pz-CP2	delta	0.28 ± 0.04	0.25 ± 0.04	2.79	0.042^*^
P7-CP2	delta	0.26 ± 0.03	0.24 ± 0.04	2.95	0.029^*^
P7-Cz	delta	0.26 ± 0.03	0.24 ± 0.04	2.95	0.030^*^
Oz-F8	delta	0.27 ± 0.03	0.24 ± 0.04	4.52	0.001^*^
O2-F8	delta	0.26 ± 0.03	0.24 ± 0.03	2.93	0.031^*^
FC5-FC1	theta	0.22 ± 0.03	0.24 ± 0.03	−3.09	0.021^*^
Fp1-CP5	theta	0.21 ± 0.03	0.23 ± 0.03	−2.98	0.028^*^
FT9-CP5	theta	0.21 ± 0.03	0.24 ± 0.03	−2.43	0.048^*^
CP1-P8	theta	0.23 ± 0.03	0.21 ± 0.03	2.84	0.038^*^
O1-Fp2	theta	0.21 ± 0.03	0.23 ± 0.03	−2.98	0.027^*^
FT9-CP5	alpha	0.21 ± 0.04	0.23 ± 0.05	−2.68	0.048^*^
T7-CP5	alpha	0.19 ± 0.05	0.23 ± 0.05	−4.17	0.002^*^
FT9-CP1	alpha	0.20 ± 0.02	0.19 ± 0.02	3.09	0.021^*^
FC5-CP1	alpha	0.19 ± 0.03	0.21 ± 0.03	−2.61	0.032^*^
FT9-O2	alpha	0.19 ± 0.03	0.21 ± 0.04	−3.04	0.024^*^
C3-T8	alpha	0.19 ± 0.02	0.21 ± 0.03	−3.13	0.019^*^
T7-FT10	alpha	0.19 ± 0.03	0.23 ± 0.03	−5.19	0.000^*^
C4-FT10	alpha	0.19 ± 0.02	0.21 ± 0.03	−2.80	0.042^*^
P3-F8	alpha	0.19 ± 0.03	0.21 ± 0.02	−3.40	0.010^*^
FC1-Fp2	alpha	0.19 ± 0.02	0.22 ± 0.03	−2.86	0.036^*^
F3-FC1	beta	0.11 ± 0.01	0.10 ± 0.02	2.96	0.029^*^
F3-CP1	beta	0.11 ± 0.01	0.11 ± 0.01	2.68	0.027^*^
FT9-CP2	beta	0.11 ± 0.01	0.10 ± 0.01	3.80	0.004^*^
P3-CP2	beta	0.19 ± 0.03	0.21 ± 0.03	−2.61	0.032^*^
F3-FT10	beta	0.12 ± 0.02	0.10 ± 0.02	3.04	0.024^*^
FC1-FC2	beta	0.11 ± 0.01	0.10 ± 0.01	2.94	0.030^*^

*p < 0.05.

For the control group, significant differences in functional connectivity before and after the blank control were observed in the following electrode pairs: delta band: Fp1-FC5, Fz-CP1, FC5-P7, C3-Cz, Fp1-C4, P4-C4, P3-T8, P3-FT10, T7-FC6, Fp1-F4; theta band: Fp1-FC5, O1-P8, FT9-Fp2; alpha band: CP5-P3, CP1-Oz, Oz-P4, P4-FC6; beta band: FC5-CP2, Oz-FC6, FT10-F4. Detailed statistical results for these electrode pairs are presented in Table 5.

**Table 5:** Electrodes with significant differences in functional connectivity before and after training in the control group (*n* = 23, $\bar{X}$ ± S).

Connection between electrodes	Frequency band	Before training	After training	t (22)	*p*
Fp1-FC5	delta	0.24 ± 0.04	0.27 ± 0.03	−3.28	0.014^*^
Fz-CP1	delta	0.27 ± 0.03	0.25 ± 0.02	3.32	0.012^*^
FC5-P7	delta	0.24 ± 0.03	0.27 ± 0.03	−2.87	0.035^*^
C3-Cz	delta	0.25 ± 0.03	0.27 ± 0.04	−2.85	0.037^*^
Fp1-C4	delta	0.24 ± 0.02	0.26 ± 0.03	−3.15	0.018^*^
P4-C4	delta	0.25 ± 0.02	0.26 ± 0.03	−2.78	0.044^*^
P3-T8	delta	0.27 ± 0.04	0.24 ± 0.03	2.76	0.046^*^
P3-FT10	delta	0.29 ± 0.04	0.26 ± 0.04	3.02	0.025^*^
T7-FC6	delta	0.24 ± 0.03	0.26 ± 0.03	−3.53	0.008^*^
Fp1-F4	delta	0.25 ± 0.02	0.27 ± 0.03	−2.94	0.030^*^
Fp1-FC5	theta	0.22 ± 0.03	0.20 ± 0.02	2.99	0.014^*^
O1-P8	theta	0.21 ± 0.03	0.23 ± 0.02	−2.80	0.042^*^
FT9-Fp2	theta	0.22 ± 0.02	0.23 ± 0.02	−2.95	0.030^*^
CP5-P3	alpha	0.19 ± 0.04	0.23 ± 0.05	−2.94	0.030^*^
CP1-Oz	alpha	0.21 ± 0.02	0.20 ± 0.02	2.97	0.028^*^
Oz-P4	alpha	0.19 ± 0.02	0.21 ± 0.02	−2.84	0.038^*^
P4-FC6	alpha	0.19 ± 0.02	0.21 ± 0.03	−2.72	0.049^*^
FC5-CP2	beta	0.10 ± 0.01	0.11 ± 0.02	−3.45	0.009^*^
Oz-FC6	beta	0.11 ± 0.01	0.10 ± 0.01	2.81	0.041^*^
FT10-F4	beta	0.11 ± 0.01	0.10 ± 0.01	2.86	0.037^*^

*p < 0.05.

### 3.5 Correlation between EEG indicators and flight performance

To further explore the behavioral relevance of the training-induced EEG changes, Pearson correlation analyses were conducted between the EEG indicators (power spectral density and functional connectivity values) that showed significant changes after training and the flight performance metrics. The results for the training group are presented in Table 6.

**Table 6:** Correlation results between the EEG indicators and flight performance in the training group.

EEG Indicator type	Frequency band	Electrode /connection	Glide path deviation		Flight time		Flight performance	
			r	*p*	r	*p*	r	*p*
Power spectral density	alpha	CP5	−0.181	0.229	−0.337	0.022 *	0.337	0.022*
	alpha	Pz	−0.177	0.241	−0.298	0.045*	0.295	0.047*
	alpha	O1	−0.114	0.450	−0.343	0.020*	0.307	0.038*
	beta	O1	−0.072	0.634	−0.293	0.048*	0.248	0.096
Functional connectivity	delta	P7-Cz	−0.433	0.003*	−0.060	0.691	0.328	0.026*
	alpha	T7-CP5	0.000	0.998	−0.329	0.026*	0.239	0.110

*p < 0.05.

For power spectral density, significant negative correlations were observed between alpha-band PSD at electrodes CP5, Pz, and O1 and flight time (CP5: r = −0.337, *p* = 0.022; Pz: r = −0.298, *p* = 0.045; O1: r = −0.343, *p* = 0.020), indicating that higher alpha power was associated with shorter flight time. Consequently, these alpha-band PSD values showed significant positive correlations with the composite flight performance indicator F (CP5: r = 0.337, p = 0.022; Pz: r = 0.295, *p* = 0.047; O1: r = 0.307, *p* = 0.038). Beta-band PSD at electrode O1 showed a marginally significant negative correlation with flight time (r = −0.293, *p* = 0.048) but did not reach significance with the composite indicator F (*p* = 0.096).

For functional connectivity, significant negative correlations were found between delta-band functional connectivity in the P7-Cz electrode pair and glide path deviation (r = −0.433, *p* = 0.003), indicating that stronger delta functional connectivity was associated with smaller glide path deviation. This functional connectivity measure also showed a significant positive correlation with the composite indicator F (r = 0.328, *p* = 0.026). Alpha-band connectivity in the T7-CP5 electrode pair showed a significant negative correlation with flight time (r = −0.329, *p* = 0.026), suggesting that stronger alpha connectivity was associated with more efficient task execution (i.e., shorter flight time).

## 4 Discussion

This study investigates the effects of mental training on the brain functional networks of carrier-based aircraft operators from the perspectives of flight performance, power spectral density, sample entropy, and functional connectivity. The aim is to explore the impact of mental training on both flight performance and brain function during the carrier approach and landing phase. Results indicate that flight performance in the training group following mental training significantly improved compared to pre-training levels (Z = −2.859, *p* = 0.004), whereas no significant difference was observed in the control group’s flight performance (Z = −0.395, *p* = 0.693). This demonstrates that the mental training paradigm employed in this study enhances participants’ performance during the landing approach manoeuvre. Statistical analysis was conducted on the deviations from the standard glide path following training for the training group and after the blank control for the control group. Significant differences were found between the two groups (U = 167.000, Z = −2.143, *p* = 0.032). The median values indicated that the deviation from the standard glide path during the training group’s post-training glide landing was smaller than that of the control group after the blank control. This demonstrates that the training paradigm employed in this study enhances both the stability and precision of the operators’ manoeuvre. Statistical analysis of flight times post-training for both the training group and control group revealed that the training group exhibited significantly shorter flight time during the post-training glide landing maneuver compared to the control group (U = 72.000, Z = −4.229, *p* = 0.000). Consequently, the training protocol also demonstrated a marked effect in enhancing the operational speed of personnel.

Regarding changes in brain functional states within the training group before and after mental training, from the perspective of EEG PSD, the results indicate that post-training, compared to pre-training, the PSD in the alpha band of the frontal, parietal, and occipital regions, the theta band of the central region, and the beta band of the occipital region all increased significantly (*p* < 0.05). Conversely, the control group showed no significant differences in PSD across any frequency bands before and after mental training. This demonstrates that the training’s impact on participants’ brain activity involves multiple brain regions and various frequency bands. Researches indicate that by increasing alpha power in regions associated with processing information, irrelevant details can be filtered out, thereby directing attention (Jensen and Mazaheri, 2010). Some studies also suggest that the alpha band may regulate attention by limiting the flow of both relevant and irrelevant information through the suppression of neuronal discharge rates (Haegens et al., 2011). The increase of PSD in alpha-band observed in this study may reflect participants’ enhanced ability to selectively focus on external information directly relevant to flight performance after mental training, thereby suppressing less relevant or irrelevant inputs (Van Diepen et al., 2019). Results indicate increased alpha band power in the occipital cortex of the left hemisphere. We hypothesize that by training participants to selectively ignore instrument information on the contralateral side of the training system interface through visual attention, they performed the carrier landing approach maneuver primarily guided by the alignment angle instrument. Power in the alpha band increases in the parietal cortex of the left hemisphere, potentially indicating selective inhibition of contralateral hand manipulation tasks. This prevents excessive local movement of the joystick, thereby preserving control precision. After mental training, the PSD in the central theta band increased, indicating that training elevated the working memory load during task performance to achieve higher task performance. Additionally, research indicates a positive correlation between power in the occipital beta band and visual attention (Gola et al., 2012). In this study, the beta band power in the occipital region was higher after training than before, indicating that mental training enhanced the visual attention of the participants.

Sample entropy reflects the complexity of EEG time series. This study found that for brain region activity sample entropy, the brain regions showing significant differences between the training group and the control group before and after mental training were markedly different. Changes in sample entropy for the training group were primarily concentrated in the theta band of the central and parietal regions, while those for the control group were mainly concentrated in the alpha band of the central and parietal regions. Theta band activity is closely associated with working memory load. The increase in sample entropy of theta band signals following training indicates heightened complexity in EEG signals during task performance. Enhanced brain activity related to the task may represent the effect of mental training. However, the control group exhibited reduced alpha-band sample entropy following the blank control task, indicating decreased complexity in EEG signals during the task. Therefore, we hypothesize that mental training influences neural oscillatory activity in the central and parietal regions of the brain, and this effect may be associated with improved flight task performance.

The carrier landing approach task involves a complex cascade of perception, judgment, decision-making, and control that demands precise inter-regional brain coordination for a successful landing. Therefore, this study compared and analyzed the differences in functional connectivity between brain regions in the training group and the control group before and after mental training. Research findings indicate that in the training group, the connectivity strength across all frequency bands in the left hemisphere of the brain was slightly higher than that in the right hemisphere. Furthermore, the interactive activity between brain regions across all frequency bands was greater than in the control group, particularly in the delta and alpha frequency bands. After mental training, functional connectivity in the delta band across all electrodes was reduced in the training group compared to pre-training levels, whereas the control group primarily exhibited increased connectivity post-control compared to pre-control levels. The alpha band showed more connections with functional connectivity differences in the training group than in the control group, and functional connectivity in all electrodes of the training group was greater after mental training than before. The differences observed in the delta and alpha bands between the training and control groups may be attributable to the mental training. A total of 13 pairs of connections showed significant differences in the delta band before and after mental training, concentrated in the frontal–parietal, frontal-temporal, frontal-occipital, occipital-parietal, and parietal-central regions. The frontal lobe exhibits the most extensive connections with other brain regions. This pattern is consistent with the engagement of the frontoparietal control network, which is known to support attention, working memory, and decision-making during complex task performance (Marek and Dosenbach, 2018). The carrier landing approach task requires the operator to continuously adjust movement control based on perceived spatiotemporal states until a safe landing is achieved, necessitating close coordination between the frontal lobe and parietal, occipital, and temporal regions (Mohanavelu et al., 2020). The training group exhibited 10 pairs of connections with significant differences in alpha band activity before and after mental training, observed in the frontal–parietal, frontal-temporal, frontal-occipital, frontal-central, and temporal-central regions. Recent longitudinal studies on cognitive training have shown that training-induced improvements in task performance are accompanied by increased alpha-band functional connectivity. Ericson et al. (2024) demonstrated that strengthening of alpha synchronization is a neural correlate of cognitive transfer following training. Similarly, strong connections between the frontal lobe and other brain areas were evident. Evidently, the training significantly influenced interactions between brain regions, enhancing their collaborative functioning. Consequently, the significantly improved performance post-training compared to pre-training may be attributed to these altered brain interactions.

In summary, the mental training paradigm employed in this experiment significantly improved the stability, accuracy, and task completion time of participants’ carrier landing approach maneuvers (*p* < 0.05). Simultaneously, significant differences (*p* < 0.05) were observed between the training group and the control group in the PSD, sample entropy, and functional connectivity. Therefore, providing carrier-based flight trainees with specialized mental training in emotional stability, attention allocation, spatial-distance perception, and orientation perception before carrier landing approach training can improve flight performance and sustain flight stability and precision. Moreover, the EEG findings reveal specific cortical areas exhibiting significant potential changes before and after mental training, suggesting that these regions may be promising targets for future intervention studies aimed at enhancing flight performance.

Several limitations of this study should be acknowledged. First, each landing approach lasted approximately 3–4 min, limiting the number of trials per session to one. While this minimized fatigue and preserved ecological validity, it precluded trial averaging and assessment of within-session reliability. Future studies could incorporate multiple landings with rest intervals to further validate the stability of the observed effects. Second, the mental training program combined cognitive and motor tasks, making it impossible to isolate the specific contributions of each component. Although our primary goal was to evaluate the integrated training package as it might be applied in practice, future research employing component-specific designs is needed to identify the neural mechanisms underlying training-induced plasticity.

## Data Availability

The raw data supporting the conclusions of this article will be made available by the authors, without undue reservation.
